# The Effect of Breed, Gender, and Acid Stimulation in Dog Saliva Proteome

**DOI:** 10.1155/2018/7456894

**Published:** 2018-06-03

**Authors:** Sónia Lucena, Ana V. Coelho, Fernando Capela-Silva, Asta Tvarijonaviciute, Elsa Lamy

**Affiliations:** ^1^Instituto de Ciências Agrárias e Ambientais Mediterrânicas (ICAAM), Universidade de Évora, 7000-083 Évora, Portugal; ^2^Departamento de Medicina Veterinária, Escola de Ciências e Tecnologia, Universidade de Évora, 7000-083 Évora, Portugal; ^3^Instituto de Tecnologia Química e Biológica António Xavier, Universidade Nova de Lisboa, 2780-157 Oeiras, Portugal; ^4^Departamento de Biologia, Escola de Ciências e Tecnologia, Universidade de Évora, 7000-671 Évora, Portugal; ^5^Interdisciplinary Laboratory of Clinical Analysis (Interlab-UMU), Regional Campus of International Excellence “Campus Mare Nostrum”, University of Murcia, 30100 Espinardo, Murcia, Spain

## Abstract

Saliva gained interest as a potential noninvasive source of biomarkers in humans and that interest starts to be extended also to other animal species. For this purpose, the knowledge of the salivary proteome in healthy conditions and the factors that affect it and how they affect it are necessary. The aim of the present study was to assess the effect that gender and breed have in saliva proteome and the changes in it induced by stimulation with acid. Saliva from 4 different purebred dogs (Portuguese Podengo, Greyhound, Rafeiro Alentejano, and Beagle) of both genders was collected without and after stimulation with lemon juice. SDS-PAGE and two-dimensional gel electrophoresis (2-DE) profiles were compared and the proteins of interest in-gel digested and identified by mass spectrometry. Acid stimulation decreased total protein concentration and the relative amounts of some protein bands/spots. Gender appeared to have minimal effect in saliva proteome, whereas the influence of breed varies. Beagles and Portuguese Podengos were the two breeds with higher differences. In conclusion, stimulation procedures and dog breed should be considered in data analysis when using salivary proteins for diagnostic purposes.

## 1. Introduction

Physiological variables are of added value to assess the welfare and lifespan both in humans and in animals, as they provide important information for interpreting and validating emotional and biological responses, respectively [1]. Saliva has gained interest for biomarker identification, mainly due to the noninvasive nature of its collection; at the same time that it contains glandular and blood-born molecules that can change under different conditions [2]. In dogs, most of the studies have been focused on the evaluation of stress by measuring salivary cortisol levels [3]. Infectious agents, such as* Helicobacter* spp.,* Bartonella* spp., or rabies virus, have also been evaluated [4–6]. In addition, canine saliva has been used for quantification of acute phase proteins [7] and allergen measurements [8] and in forensic studies for canine mRNA determination [9]. Furthermore, recently, healthy dog saliva proteome has been characterized by shotgun proteomics, with the identification of 2,491 proteins and peptides [10]. Despite this characterization, two-dimensional electrophoresis (2-DE) salivary protein profiles of dog saliva have been less explored. Although several researchers consider that gel-based approaches provide limited information, 2-DE continues providing reliable quantitative results on differential protein expressions as they display a high number of protein species, their isoforms, and posttranslational modifications at the same time [11]. It also has the advantage of allowing modifications of the protein mixtures caused by inadequate treatment or endogenous protease activities with physiological relevance to be easily recognized via pattern disturbances by 2D gels [11].

In humans, physiological and environmental factors, such as gender, age, interindividual variability, taste stimulation, and circadian rhythms, were identified to cause differences in the human salivary protein profiles [12]. However, to the best of the author's knowledge, in dogs such influences in salivary proteome are not deeply studied. The knowledge of the possible salivary proteome changes due to different factors would later permit correcting data interpretation for disease diagnostics.

Different methods of saliva sampling in dogs have been reported in literature: (1) without stimulation [10, 13]; (2) using different stimulating methods, such as citric acid in swabs [14] or in crystals spreader in the tongue [15], beef-flavoured cotton ropes [16], dogs' snack held in front of the dog's snout [17], or visualization and smell of food [18] what could result in different salivary proteomes. Acid stimulation, which is one of the mostly used methods for stimulating saliva production in humans, has been already reported to influence human salivary proteome [12]. However, its influence in dog saliva composition has not been reported.

The aims of this study were to evaluate the possible influence of biological factors, namely, breed and gender, and different saliva sampling conditions (with and without saliva stimulation with citric acid) on dog's saliva proteome.

## 2. Materials and Methods

### 2.1. Ethical Note

Dogs used in this study belong to three kennels and to a university (University of Murcia), whose gave their informed consent and participated in the collection procedures by handling the animals. The saliva collection and all animal procedures were carried out by researcher accredited by the Federation of European Laboratory Animal Science Association (FELASA) and conformed to legislation.

### 2.2. Dog Population

Dog population for each breed by gender and age is shown in Table 1. All were healthy and normal weight animals. Only male's pure breed Beagle were neutered animals.

### 2.3. Saliva Collection

Saliva samples were collected in the afternoon between 3:30 and 6:30 pm. Dogs did not eat for 16–18 hours prior to saliva sampling. Water was provided ad libitum. Saliva was collected by rolling a cotton cylinder (Salivette®, Sarstedt) inside each dog's mouth as described previously [19, 20]. The cotton cylinders were inserted under the dog's tongue for chew, until completely soaked with saliva, for a maximum of two minutes [21]. Two to three sample were collected in all animals, in different days. In one of these sample collections two to three drops of lemon juice were put under the tongue for stimulating saliva flow (acid stimulation). Only for Rafeiro Alentejano breed acid stimulated saliva collection was not possible. After collection, the cotton cylinders were immediately placed on ice, until laboratory arrival, which lasted no more than 30 minutes. In the laboratory saliva was extracted from the cotton roll by centrifugation at 4°C, at 5000 rpm, for 5 min, and immediately stored at −20°C for further analysis.

### 2.4. Total Protein Concentration

Bradford method protein assay [22] with BSA as the standard protein (Pierce Biotechnology, Rockford, IL, USA) was performed to determine the total protein concentration of each sample. Standards and samples were run in triplicate, in 96-well microplates. Absorbance was read at 600 nm in a microplate reader (Glomax, Promega).

### 2.5. SDS-PAGE

Proteins from individual saliva samples of all animals (both without and with acid stimulation) were separated by SDS-PAGE electrophoresis in 14% acrylamide gels in a mini-protean apparatus (BioRad) as described before [23]. Briefly, a total of 15 *μ*g protein from each saliva sample was run in each lane. The samples were resuspended in sample buffer [Tris–HCl 0.125 M pH 6.8, 2% (w/v) SDS, 5% (v/v) 2-mercaptoetanol, 20% (v/v) glycerol traces of bromophenol blue], heated at 95°C for 5 minutes, and run at a constant voltage of 140 V until the dye front reaches the end of the gel. Gels were fixed in 40% methanol, 20% acetic acid, for one hour, stained with Coomassie Brilliant Blue (CBB) G-250 (0.125% CBB G-250, 20% ethanol) for two hours and destained in several washes with distilled water. A scanning Molecular Dynamics densitometer with internal calibration and LabScan software (GE, Healthcare) were used to acquire gel images and to determine the percentage of volume of each protein band; GelAnalyzer software (http://www.gelanalyzer.com/) was used to analyze the gel images. Molecular masses were determined in accordance with molecular mass standards (Bio-Rad Precision Plus Protein Dual Color 161–0394) run with protein samples.

### 2.6. Two-Dimensional Gel Electrophoresis (2-DE)

#### 2.6.1. Protein Precipitation

Due to the limited amount of individual saliva samples, the unstimulated and acid stimulated saliva samples from each breed and gender were mixed in pools, constituting a total of 12 pools: (1) unstimulated female Portuguese Podengo; (2) unstimulated male Portuguese Podengo; (3) stimulated female Portuguese Podengo; (4) stimulated male Portuguese Podengo; (5) unstimulated female Greyhound; (6) unstimulated male Greyhound; (7) stimulated female Greyhound; (8) stimulated male Greyhound; (9) unstimulated female Rafeiro Alentejano; (10) unstimulated male Rafeiro Alentejano; (11) unstimulated male Beagle; (12) stimulated male Beagle. Volumes of saliva from each pool containing 250 *μ*g of total protein were used. The volume of each pool was mixed with equal volume of TCA 20% (m/v), incubated overnight, at −20°C, followed by centrifugation at 15,000*g*, 30 min, and two cold-acetone washes. This protocol as previously observed by us allows satisfactory results for preparation of dog saliva samples for 2-DE [24].

#### 2.6.2. 2-DE Protein Separation

For 2-DE, the precipitates were mixed with 250 *μ*L rehydration buffer [7 M urea, 2 M thiourea, 4% (w/v) CHAPS, 2% (v/v), 60 mM DTT and traces of bromophenol blue] +5 *μ*L IPG buffer +5 *μ*L NaOH. Then the precipitates were sonicated until total resuspension and incubated during 1 h at room temperature, being subsequently centrifuged for 5 min at 10000 rpm. IPG strips (13 cm, pH 3–10 NL; GE, Healthcare) were passively rehydrated overnight with this solution. Focusing was performed in a Multiphor II (GE, Healthcare) at 20°C, with the programme (gradient): (1) 0–300 V for 2 h; (2) 300 V for 2 h; 300 V to 3500 V for 6 h; 3500 V for 6 h. Focused strips were equilibrated in two steps of 15 min each with equilibration buffer [50 mM Tris–HCl, pH 8.8; 6 M urea; 30% (v/v) glycerol and 2% (w/v) SDS], with the addition of 1% (w/v) DTT and 65 mM iodoacetamide in the first and second steps, respectively. After equilibration the strips were applied in the top of a SDS-PAGE gel 14% acrylamide and run at 150 V constant voltage in a mini-protean system (BioRad). Staining with CBB-G250 and destaining were done through the same protocol described for SDS-PAGE gels. Gel images were acquired using the same scan method and apparatus described for SDS-PAGE gels. ImageMaster 2D Platinum v7 software was used to analyze these gel images. Spot editing and the match were performed automatically and corrected manually. Spot volume was normalized to the total spot volume. Three laboratorial replicates of each pool were run.

### 2.7. Protein Identification

Bands and spots that differed among the factors tested were manually excised from gels and digested with trypsin following the protocol already described [25]. MALDI-TOF/-TOF mass spectrometry was used for protein identifications. Tryptic peptide mixtures were acidified with 5% (V/V) formic acid, desalted, and concentrated using home-made reversal phase (R2 pores-Applied Biosystems) microcolumns (R2 pores-Applied Biosystems). Peptides were eluted with the matrix solution (*α*-cyano-4-hydroxycinnamic acid Fluka) 5 mg/mL in 50% (v/v) acetonitrile and 5% (v/v) formic acid. MS and MS/MS data were acquired in positive reflector mode in a 4800 Plus AB SCIEX using the software 4000 Series Explorer, version 3.5.3.3 (Applied Biosystems).

Peptide mass spectra were acquired using a MALDI-TOF/TOF 4800 plus MS/MS (Applied Biosystems® Life Technologies, Carlsbad, United States of America). Data were acquired in positive MS reflector using a PepMix1 (LaserBio Labs, Sophia-Antipolis, France) to calibrate the instrument. Each reflector MS spectrum was collected in a result independent acquisition mode, using 750 shots per spectra in 800–4000 *m*/*z* range and fixed laser intensity to 3100 V. Fifteen of the strongest precursors were selected for MS/MS. MS/MS analyses were performed using CID (Collision Induced Dissociation) assisted with a collision energy of 1 kV and a gas pressure of 1 × 10−6 Torr. For each MS/MS spectrum, 1400 laser shots were collected, using fixed laser intensity of 4400 V. Processing and interpretation of MS and MS/MS spectra were performed with the 4000 Series Explored™ Software (Applied Biosystems® Life Technologies, Carlsbad, United States of America).

Protein identification was performed using MS and MS/MS spectral data and ProteinPilot (Applied Biosystems, version 3.0, rev. 114732) on Canis canis database (85118 sequences; 46,697,962 residues) retrieved from NCBI (downloaded in October 2017). Searches included trypsin as digesting enzyme; peptide mass tolerance of 50 ppm; fragment mass tolerance of 0.5 Da and possible oxidation, carbomidomethylation, or deaminidation as variable amino acid modifications with one missed cleavage. Peptides were only considered if the ion score indicated extensive homology (*p* < 0.05). Proteins were considered if the protein score indicated significant statistical confidence (*p* < 0.05). Protein identifications with only one matched peptide were considered if they were identified with >95% confidence.

### 2.8. Statistical Analysis

Multivariate analyse of protein bands, on one hand, and protein spots, on the other, were performed with MetaboAnalyst 3.6 to evaluate clustering of individuals or groups [26]. Data normalization was used when normal distribution was not observed, using transformation (log10) or scaling methods, alone or combined. The method chosen was the one that allowed data to be normally distributed. For univariate analysis, *t*-test, one-way ANOVA, and two-way ANOVA were used for comparison of protein profiles (band percentage volume or spots percentage volume) between unstimulated and acid stimulated saliva and among breeds and genders. For Multivariate Analysis, partial least squares discriminant analysis (PLS-DA) was used. Discriminant variables selection was done using variable importance in the projection (VIP) with a threshold of 1.0. Finally, paired-samples *t*-test was used for comparison of total protein concentration between saliva samples with and without stimulation. Statistical significance was considered for *p* < 0.05.

## 3. Results

### 3.1. Effect of Acid Stimulation on Salivary Proteome

#### 3.1.1. Total Protein Concentration

Total protein concentration decreased significantly in stimulated saliva in males of both pure breeds Portuguese Podengo and Beagle. In females, no statistically significant differences were observed for saliva collected under the two conditions (Table 2). Concerning salivary flow rate, although this was not measured, it was possible to observe a tendency for higher salivary flow rates in big, comparatively to small breeds and higher salivary flow rate after lemon juice induction, in all breeds.

#### 3.1.2. SDS-PAGE Profile

**Figure 1:**
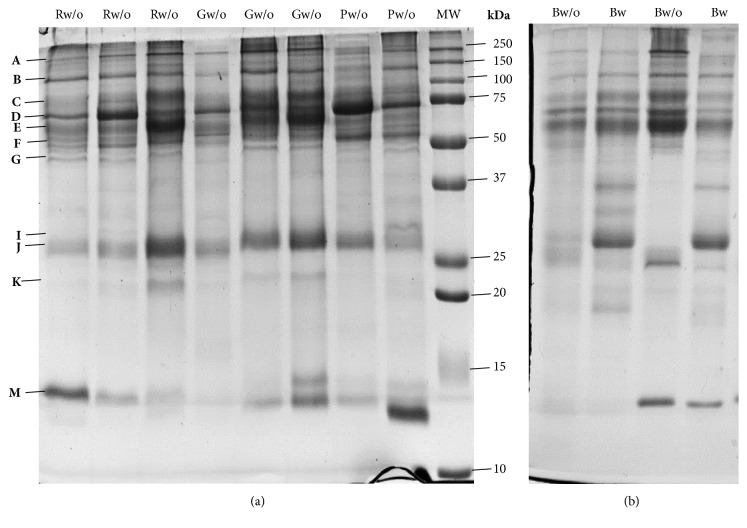
Representative SDS-PAGE profile of dog saliva: (a) from different breeds without stimulation (R: Rafeiro; G: Greyhound; P: Portuguese Podengo); (b) from Beagles without (w/o) and with (w) acid stimulation; MW: molecular mass marker; upper letters indicate the different protein bands.

Among the 16 protein bands, with molecular masses between 20 and 245 kDa, observed in SDS-PAGE protein profiles (Figure 1), some presented changes in their intensities/volumes, which were induced by acid stimulation. Some of these changes were observed to be dependent on the dogs' breed and/or gender. Considering the total of the animals, 2 of the protein bands decreased (F and J) and one increased (I1) with acid stimulation (Table 3). Concerning bands F and J, the decreased levels were observed only in males and not in females.

By considering the dog breeds separately, changes induced by stimulation were observed only for Beagles: decreased expression levels of 4 protein bands (B, D, F, and J) and increased expression level of 1 protein band (I1) (Table 3). Information about mass spectrometry details of identified proteins is present in Table 4.

**Figure 2:**
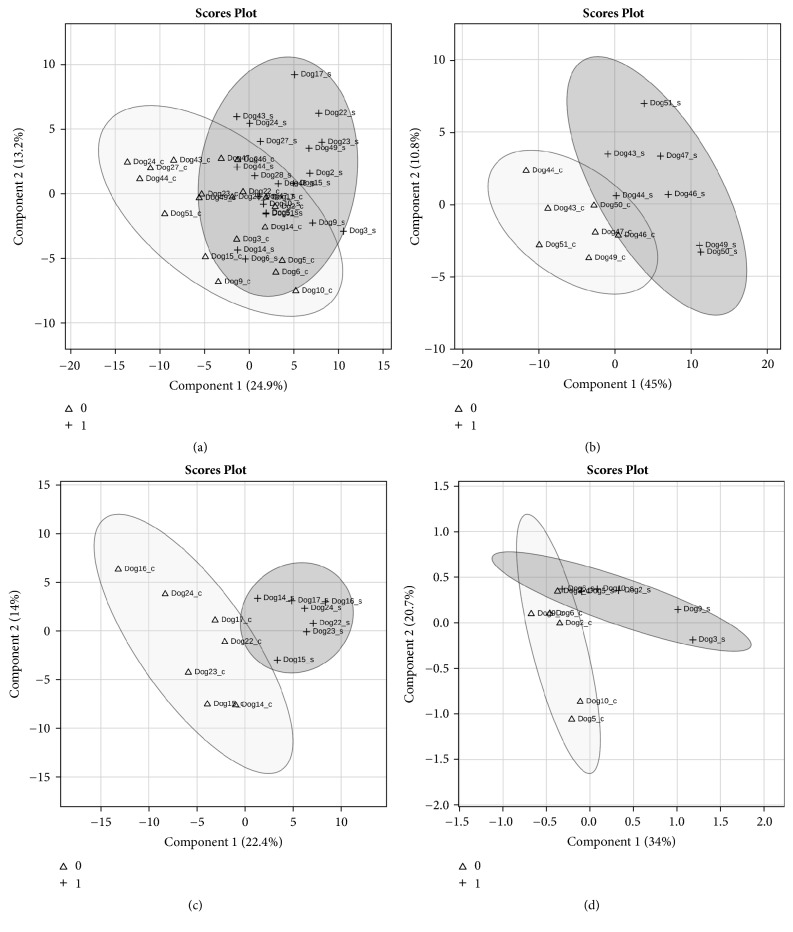
PLS-DA of saliva samples SDS-PAGE bands for all dogs (a), Beagles (*n* = 7) (b), Greyhounds (*n* = 7) (c), and Portuguese Podengos (*n* = 6) (d). Scaling was applied to rows when needed; *X* and *Y* axes show principal component 1 (PC1) and principal component 2 (PC2), respectively, and the total variance explained by each of them. Δ: with acid stimulation; +: without stimulation.

Although, in the pure breeds Portuguese Podengo and Greyhound, none of the individual bands from SDS-PAGE protein profiles showed statistical significant intensity differences, between the saliva collected with and without acid stimulation, the multivariate PLS-DA model clustered separately unstimulated saliva from acid stimulated saliva, in these two breeds (Figure 2). The protein bands J, K, and M were the major contributors for the differences in Greyhounds. Band M was identified as containing full-double-headed protease inhibitor, whereas the other two bands resulted in no confident identification. The protein bands C, E, and G, identified as containing IgGFc-binding protein and serum albumin, were the major contributors for differences in Portuguese Podengos (Supplementary Figure 1).

#### 3.1.3. Two-Dimensional Protein Profile (2-DE)

**Figure 3:**
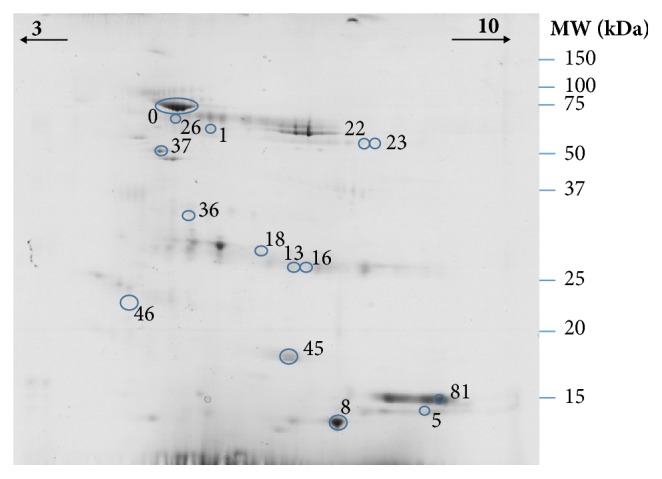
Representative dog's saliva 2-DE gel. Spots excised for digestion and identification by MS are numbered.

By analyzing 2-DE salivary protein profiles (Figure 3), 3 protein spots were observed to be present in lower volume in the saliva collected after stimulation: spot 0 (34.4 ± 5.14 and 13.9 ± 2.97% vol., saliva without and with stimulation, respectively), spot 5 (0.73 ± 0.02 and 0.47 ± 0.05% vol., saliva without and with stimulation, respectively) and spot 81 (0.5 ± 0.24 and 0.21 ± 0.20% vol., saliva without and with stimulation). These spots were identified as serum albumin subunit A, cytoskeletal keratin, and one unknown protein (Table 5).

### 3.2. Effect of Dog's Breeds and Genders on Salivary Proteome

#### 3.2.1. Total Protein Concentration

The four different breeds did not differ among them for the total protein concentration of saliva, as shown by univariate statistical analysis. Also, no differences were observed between genders, neither for saliva collected without nor saliva collected with acid stimulation.

#### 3.2.2. SDS-PAGE Profile

Salivary protein profiles of the 4 dog breeds studied were compared for the saliva collected without acid stimulation. Six protein bands showed a different volume among dog breeds (Table 6): bands containing serum albumin were observed to be increased in Beagles, whereas a band containing a full-double-headed protease inhibitor was decreased, comparatively to the other breeds; bands containing albumin and IgGFc-binding protein were increased and one not identified was decreased in Portuguese Podengo. No trends for gender were found and no relationship between breed and gender was found, as well.

**Figure 4:**
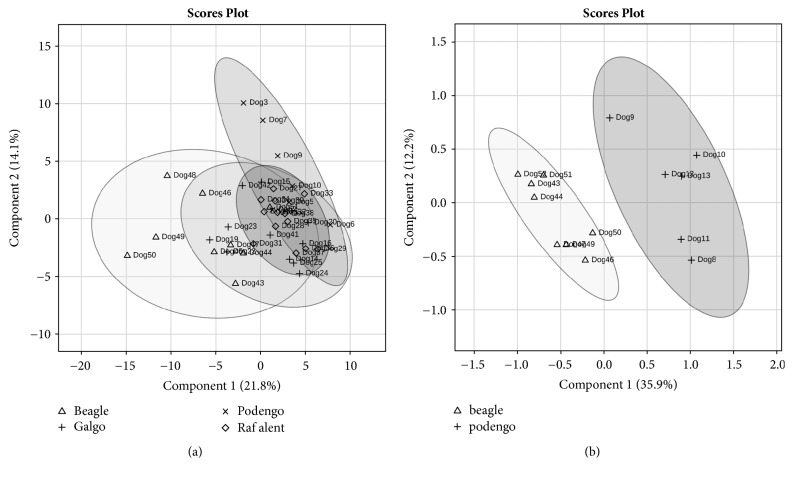
Partial Least Square Determinant Analysis (PLS-DA) model for all dog unstimulated saliva samples SDS-PAGE bands [Δ: Portuguese Podengo (*n* = 7); +: Greyhound (*n* = 11); ◊: Rafeiro Alentejano (*n* = 13); and x: Beagles (*n* = 10)] (a) and for stimulated saliva samples SDS-PAGE bands [+: Portuguese Podengo (*n* = 6) and Δ: Beagles (*n* = 8)] (b). Scaling was applied to rows when needed; *X* and *Y* axes show principal component 1 (PC1) and principal component 2 (PC2), respectively, and the contribution of each of them for explaining the total variance.

Through the multivariate PLS-DA model, that has into account the interrelationship among variables, it was possible to cluster Portuguese Podengos and Beagles more distant, comparatively to the other breeds (Figure 4(a) and Supplementary Figure 2). The differences between Portuguese Podengo and Beagles for nonstimulated saliva were confirmed in the saliva collected after acid stimulation, by univariate analysis. In this case, it was also possible to observe that these breeds differ in saliva protein profile, with five proteins bands (E, F, I1, J, and M) observed to be differently expressed (Table 7 and Figure 4(b)).

#### 3.2.3. 2-DE Saliva Profile

2-DE salivary protein profiles of the several dog breeds evaluated presented differences in the percentage volumes of 7 protein spots. Through ANOVA (univariate analysis) it was observed that Portuguese Podengo presented higher levels of 5 salivary protein spots [1 (*p* = 0.034), 18 (*p* = 0.041), 22 (*p* = 0.046), 36 (*p* = 0.036), and 46 (*p* = 0.014)], comparatively to the other breeds. Among them, only spot 18 was identified (as a light-chain of immunoglobulin lambda-1). Spots 8 (*p* = 0.043) and 26 (*p* = 0.015) were present at different levels in Beagles, the spot 8 (identified as double-headed protease inhibitor) being in lower levels than in Greyhounds and the spot 26 (not identified) in higher levels than in the other breeds.

**Figure 5:**
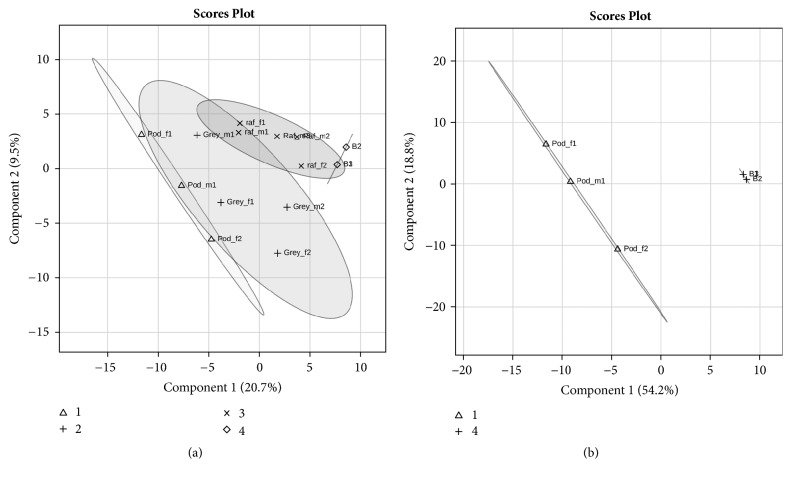
PLS-DA of dog saliva pool samples 2-DE spots of each breed (a) or considering only Portuguese Podengo and Beagles (b). Log transformation was applied to rows; *X* and *Y* axis show principal component 1 (PC1) and principal component 2 (PC2), and the respective % of explanation for the total variance. 1 – Portuguese Podengo; 2- Greyhound; 3- Rafeiro Alentejano; 4- Beagle.

Besides these spots, multivariate PLS-DA model clustered Portuguese Podengo distinctly from Beagles and Rafeiro Alentejano breeds in 2-DE protein profiles (Figure 5). Spots 1, 18, 23, 45, and 82 were the ones that most contributed to these differences (Supplementary Figure 3). Detailed information about MS/MS identification of the referred spots is presented in Table 5.

In the case of spots 45 and 81, the identification resulted in unknown proteins. However, through BLAST analysis, it was possible to observe 83% homology between the protein present in spot 45 and a S100 calcium binding protein A9 and 83% homology between the protein present in spot 81 and keratin 8.

## 4. Discussion

In this study, the influence of gender and acid stimulation on the normal dog salivary proteome of different breeds was studied through in-gel based proteomics approach. For all the breeds, animals with a wide range of ages were included in the study. The number of proteins observed and identified in dog saliva through this methodology is much lower than the one reported in other studies, using LC-MS/MS [10, 27]. Nevertheless, in this study, dog gel protein profiles presented what can be of utility for studies where protein isoforms and/or posttranslational modifications (PTMs) are of interest [11]. SDS-PAGE and 2-DE protein separations were simultaneously performed in this study due to the limited amount of individual saliva. As such, SDS-PAGE was used for assessing variability and to make comparisons using individual information. Since this approach only allows separation according to molecular masses, several proteins must be present in each band, making it difficult to know the one (or several) responsible for changes. 2-DE profiles of saliva pools were used to add such detail.

No significant differences among breeds or between genders were observed on total protein concentration of normal dog's saliva. However, a decrease in the total protein concentration after acid stimulation was observed, especially in males of both pure breeds Portuguese Podengo and Beagle. In terms of profiles, proteins such as cytoskeletal keratin, serum albumin, and IgGFc-binding proteins were identified in bands and/or spots whose levels decreased with acid stimulation. IgG Fc-binding protein has been recently identified as one of the more abundant proteins in dog saliva [10] being a protein involved in binding IgG on mucosal surfaces [28]. To our knowledge, there are no other reports, in the literature, concerning the effect of acid stimulation on salivary proteome of dogs or other animals. But, our results are in accordance with studies performed in humans [12], where it was observed that acid stimulation produced considerable major changes, namely, in proteins related to immune function, inflammation, and cell movement [12]. Also Lorenz et al. (2011) [29] observed significant decreases on the relative abundance of several protein spots, in human saliva, after citric acid stimulation. It is curious that keratin is a protein from the cytoskeleton and IgG Fc-binding protein is a gel-like component of the mucosa. Stimulation with lemon juice raised the total volume of saliva produced and, as such, the cotton roll needed less time in the mouth for getting enough saliva amounts. Such decreased time of saliva collection, associated with fewer movements, may have resulted in a lower incorporation of components from the epithelium in the samples. In fact, the possibility of variations in the levels of these proteins being done to this effect was recently suggested [13].

In dogs, saliva collection without stimulation has the constraint of allowing obtaining only limited volumes of saliva for performing some laboratorial techniques [30]. However, if stimulation is needed it is important to have in mind the referred differences in protein composition that such stimulation is producing.

In the present study, we could observe that salivary protein composition varies among different dog breeds, but no major differences were observed between genders. The reduced impact of gender in dog salivary proteome observed is in agreement with others recently published [13]. Our results go in accordance with these observations.

Two of the breeds that most differed between them were Portuguese Podengo and Beagle. According to Federation Cynologique Internationale (FCI) (http://www.fci.be, accessed on January 31, 2018) purebred Portuguese Podengo is a primitive type of breed, hunting dog probably originating from the ancient dogs, traditionally used for helping in rabbit or birds hunting, but without working trial [31]. This breed is also used as a watch and companion dog. Despite being a pure breed, it is expected that individuals present higher genetic variability than Beagles, since this last has been bred in a controlled way, also for use in laboratory studies. Also according to FCI, purebred Beagles belong to a small-sized hound group with working trial. By using clustering analysis, to define phylogenetic tree, this breed belongs to a cluster comprised mostly by modern breeds used in hunting [32].

Bands containing chains of canine serum albumin and IgGFc-binding protein were proteins differently expressed among dog's breeds. One of the proteins observed to be present in lower amounts in Beagles, both in SDS-PAGE and in 2-DE protein profiles, was the full-double-headed protease inhibitor from the submandibular glands. This protein is a serine type endopeptidase, which has been assumed to protect mucosal cells in mouth and oesophagus against the action of proteinases from microbial origin and/or ingested with food [33].

In the present study only a limited number of proteins were observed to differ with stimulation and/or among breeds. Even some protein spots failed a positive identification, which can be related to a lower number of proteins present in curated protein databases, comparatively to other species, such as humans. On the other hand, in this study, dogs were available from pure breed kennels and some of the differences observed for the different breeds can be done to different types of dog food consumed. Further studies, with a higher number of animals per breed, higher number of breeds and controls for type of food, and other treatments are necessary to have a better characterization of each breed saliva proteome.

## 5. Conclusions

This work, in line with what was hypothesized, allowed us to conclude that dog salivary protein composition is influenced by different factors. Despite the need of procedures that allow the collection of higher amounts of saliva, it is necessary to be aware that techniques such as acid stimulation not only induce higher salivary flow rates, but also change the levels/proportion of various salivary proteins. It is also of interest to retain that dog salivary proteome should be considered according to dog breed, since this was observed to be a factor responsible for variations in the proportion of different salivary proteins. In fact, breed appears to have even more influence than gender. Nevertheless, that does not mean that gender should be ignored, in dog saliva analysis. Despite males and females presenting minimal differences in salivary profiles, in this study differences in the way each gender responded to stimulation were observed.

From our knowledge this is one of the first studies evaluating factors affecting dog saliva electrophoretic protein profiles. More studies are needed to increase the knowledge about dog saliva proteome, in order to use it in research and diagnosis.

## Figures and Tables

**Table 1:** Dog population for each breed by gender and age.

Breed	Average body weight (Kg)	Age (years)	Gender	
			Female	Male
Portuguese Podengo	4-5	0.5–10	7	6
Greyhound	26–40	1–7	9	6
Rafeiro Alentejano	35–50	0.5–8	8	7
Beagle	9–11	2–11	0	10

**Table 2:** Comparison of total protein concentration (mean ± standard error) between saliva with acid stimulation and saliva without stimulation, for each dogs breed and gender.

	Total protein concentration (*µ*g/mL)		
	with acid stimulation	without stimulation	*p*
Breed			
Portuguese Pondego (*n* = 6)	843.0 ± 163.6	2385.7 ± 482.9	0.036^*∗*^
Greyhound (*n* = 7)	961.7 ± 72.3	1146.7 ± 504.7	0.354
Beagle (*n* = 7)	1273.3 ± 161.8	1811.8 ± 246.3	0.033^*∗*^
Gender			
Female (Podengo, *n* = 4, Greyhound, *n* = 4)	950.1 ± 115.3	1743.3 ± 404.3	0.112
Male (Beagle, *n* = 7, Podengo, *n* = 2, Greyhound, *n* = 3)	1049.2 ± 110.5	1737.3 ± 170.7	0.001^*∗*^

^*∗*^Statistically significant differences for *p* < 0.05.

**Table 3:** Protein bands differently expressed (mean ± standard error) between saliva collected with and without acid stimulation.

Bands	% vol		*p*
	Without acid stimulation	With acid stimulation	
Total of animals (*n* = 20)			
F	8.26 ± 0.46	5.61 ± 0.58	0.002^*∗*^
I1	3.69 ± 0.51	7.62 ± 1.01	0.002^*∗*^
J	12.81 ± 0.54	9.24 ± 0.63	0.0008^*∗*^

Beagles (only males) (*n* = 7)			
B	9.10 ± 0.79	6.13 ± 0.39	0.004^*∗*^
D	10.28 ± 1.04	6.9 ± 0.39	0.004^*∗*^
F	8.34 ± 0.91	4.15 ± 0.64	0.002^*∗*^
I1	3.79 ± 1.00	10.10 ± 1.6	0.010^*∗*^
J	11.88 ± 0.98	8.43 ± 0.70	0.002^*∗*^

Males (three breeds) (*n* = 12)			
F	8.34 ± 0.54	5.29 ± 0.69	0.003^*∗*^
J	12.88 ± 0.68	8.56 ± 0.79	0.0003^*∗*^

^*∗*^Statistically significant differences for *p* < 0.05.

**Table 4:** Mass spectrometry identification of proteins present in bands from saliva SDS-PAGE profiles.

Band	Protein	NCBI Accession Code Accession n	Estim/theoret MW (kDa)^#^	ID Score^*∗*^	Seq. Cov. (%)	Matched Peptides MS (MS/MS)
A	Mucin-19	XP_022267206.1	240.6/340.8	201	11	21 (5)
C	IgGFc-binding protein	XP_022261796.1	75/318.0	187	14	14 (9)
D	Chain A, Crystal Structure Analysis Of Canine Serum Albumin	pdb|5GHK|A	67.8/65.7	815	52	15 (11)
E	Serum albumin isoform X1	XP_005628024.1	61.3/68.6	661	44	12 (10)
F	IgGFc-binding protein	XP_022261796.1	52.6/318.0	313	8	13 (6)
M	Full-double-headed protease inhibitor, submandibular gland	sp|P01002.1|IPSG_CANLF	12.2/12.8	166	46	6 (3)

^#^MW values observed in gel versus theoretical ones. ^*∗*^Protein score is −10*∗*log(*P*), where *P* is the probability that the observed match is a random event. Protein scores greater than 62 are significant (*p* < 0.05).

**Table 5:** Mass spectrometry identification of proteins present in spots from saliva 2-DE profiles differing between stimulation conditions and/or among breeds.

Spots	Protein	Entry reference	Estim/ theoret MW (kDa)	Estim/ theor pI	Score ID^*∗*^	% Seq. Cov.	Matched Peptides MS (MS/MS)
0	Chain A, Crystal Structure Analysis Of Canine Serum Albumin	pdb|5GHK|A (NCBI)	78.1/65.7	4.9/5.3	263	42	18 (2)
5	Keratin, type I cytoskeletal 10	Q6EIZ0 (Uniprot)	18.5/57.7	7.8/5.1	207	30	10 (5)
8	double-headed protease inhibitor, submandibular gland	XP_022264993.1 (NCBI)	17.9/15.7	6.0/8.6	428	58	4 (8)
12	Immunoglobulin J chain	XP_532398.2 (NCBI)	30.1/18.3	4.4/4.7	125	40	3 (2)
16	Immunoglobulin lambda-1 light chain isoform X34	XP_005636600.1 (NCBI)	30.0/24.8	6.0/6.4	326	33	6 (4)
18	Immunoglobulin lambda-1 light chain isoform X25	XP_022266294.1 (NCBI)	31.0/24.9	5.5/5.1	198	35	8 (4)
37	IgGFc-binding protein	XP_022261796.1 (NCBI)	59.9/318.0	4.9/5.2	267	4	7 (4)
45	Uncharacterized protein	J9P732 (Uniprot)	25.0/21.4	5.8/6.0	192	28	4 (5)
81	Uncharacterized protein	F1PW98 (Uniprot)	19.1/55.0	8.0/5.7	111	29	15 (2)

^*∗*^Protein score is −10*∗*log(*P*), where *P* is the probability that the observed match is a random event. Protein scores greater than 62 are significant  (*p* < .05).

**Table 6:** Protein bands differently expressed (mean ± standard error) between dog breeds, in saliva collected without acid stimulation.

Bands	Breed		% vol		*p*
B	Beagle	Port. Pod.	9.02 ± 0.63^a^	6.07 ± .31^b^	0.005
		Greyhound		6.71 ± .44^b^	
		Raf. Alent.		6.86 ± .38^b^	
D	Port. Pod.	Greyhound	12.59 ± 1.32^a^	8.16 ± 0.80^b^	0.005
		Raf. Alent.		8.2 ± 0.46^b^	
		Beagle		10.71 ± 0.48	
E	Beagle	Port. Pod.	13.7 ± 1.29^a^	8.14 ± 0.53^b^	0.005
		Greyhound		11.5 ± 0.98^a,b^	
		Raf. Alent.		9.97 ± 0.45^b^	
F	Port. Pod.	Greyhound	9.66 ± 0.75^a^	7.05 ± 0.62^a,b^	0.01
		Raf. Alent.		6.39 ± 0.56^b^	
		Beagle		8.53 ± 0.90^a,b^	
G	Port. Pod.	Greyhound	3.73 ± 0.72^a^	6.32 ± 0.51^a,b^	0.005
		Raf. Alent.		6.35 ± 0.35^b^	
		Beagle		8.43 ± .99^b^	
M	Beagle	Port. Pod.	1.71 ± 0.005^a^	8.64 ± 1.04^b^	0.005
		Greyhound		7.44 ± 1.25^b^	
		Raf. Alent.		5.98 ± .83^b^	

Different letters mean statistically significant differences between pairs, for *p* < 0.05. Beagle (*n* = 10); Portuguese Podengo (*N* = 7); Greyhound (*n* = 11); Rafeiro Alentejano (*n* = 13).

**Table 7:** Protein bands differently expressed (mean ± standard error) in saliva samples with acid stimulation between Portuguese Podengo  (*n* = 6) and Beagles  (*n* = 8)^#^.

Bands	% vol		*P* ^*∗*^
	Portuguese Podengo	Beagle	
E	7.39 ± 1.64	13.21 ± 0.54	0.003
F	8.89 ± 0.57	3.98 ± 0.60	9.075e − 05
I1	2.72 ± 1.07	10.64 ± 1.50	0.002
J	12.87 ± 1.21	8.40 ± 0.60	0.004
M	9.12 ± 0.68	2.85 ± 0.60	1.6369e − 05

^*∗*^Statistically significant differences for *p* < 0.05.  ^#^  *N* of Beagles used for comparison was different that the one reported in Table 3, since for 1 animal only saliva from the collection after stimulation contained enough amount for analysis, impeding that animal for being included in paired analysis reported in Table 3.

## Data Availability

The data used to support the findings of this study are available from the corresponding author upon request.

## Supplementary Materials

Supplementary 1 Supplementary Figure*    *1: PLS-DA loading plots (left) of the first two components for analysis of SDS-PAGE bands of profiles from saliva collected with and without acid stimulation in Beagles (a), Greyhound (b), and Portuguese Podengo (c); for each case, variable importance in the projection (VIP) is presented, with 1.5 score considered as thresholder (right).

Supplementary 2 Supplementary Figure*    *2: PLS-DA loading plots (left) of the first two components for analysis of protein bands of salivary profiles from the different dog breeds. Variable importance in the projection (VIP) is presented (right).

Supplementary 3 Supplementary Figure*    *3: PLS-DA loading plots (left) of the first two components for analysis of protein spots of salivary profiles from the different dog breeds. Variable importance in the projection (VIP) is presented (right).
