# Supplementary figures and images for: The Effect of Breed, Gender, and Acid Stimulation in Dog Saliva Proteome
Source: Biomed Res Int. 2018 Jun 3;2018:7456894. doi: 10.1155/2018/7456894 (PMC6008695; doi:10.1155/2018/7456894)

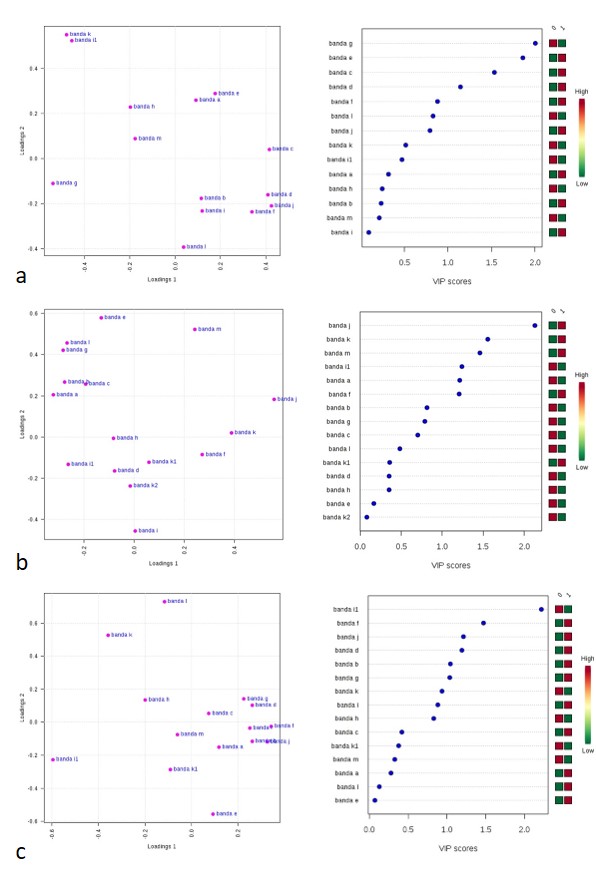

Supplement: Supplementary 1 — Supplementary Figure 1: PLS-DA loading plots (left) of the first two components for analysis of SDS-PAGE bands of profiles from saliva collected with and without acid stimulation in Beagles (a), Greyhound (b), and Portuguese Podengo (c); for each case, variable importance in the projection (VIP) is presented, with 1.5 score considered as thresholder (right). [file 7456894.f1.jpg]

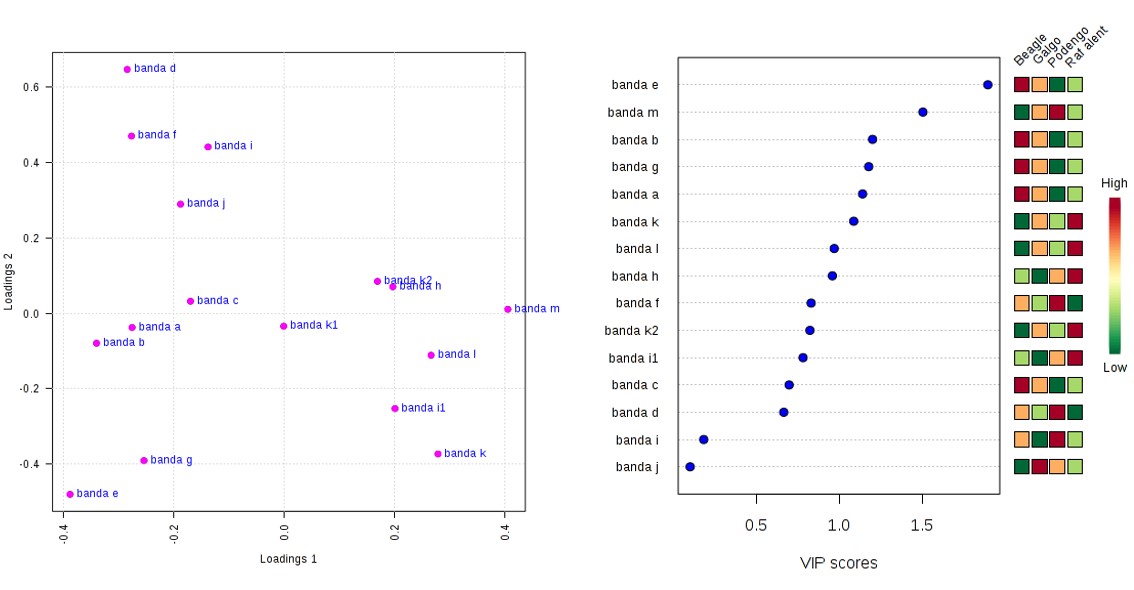

Supplement: Supplementary 2 — Supplementary Figure 2: PLS-DA loading plots (left) of the first two components for analysis of protein bands of salivary profiles from the different dog breeds. Variable importance in the projection (VIP) is presented (right). [file 7456894.f2.jpg]

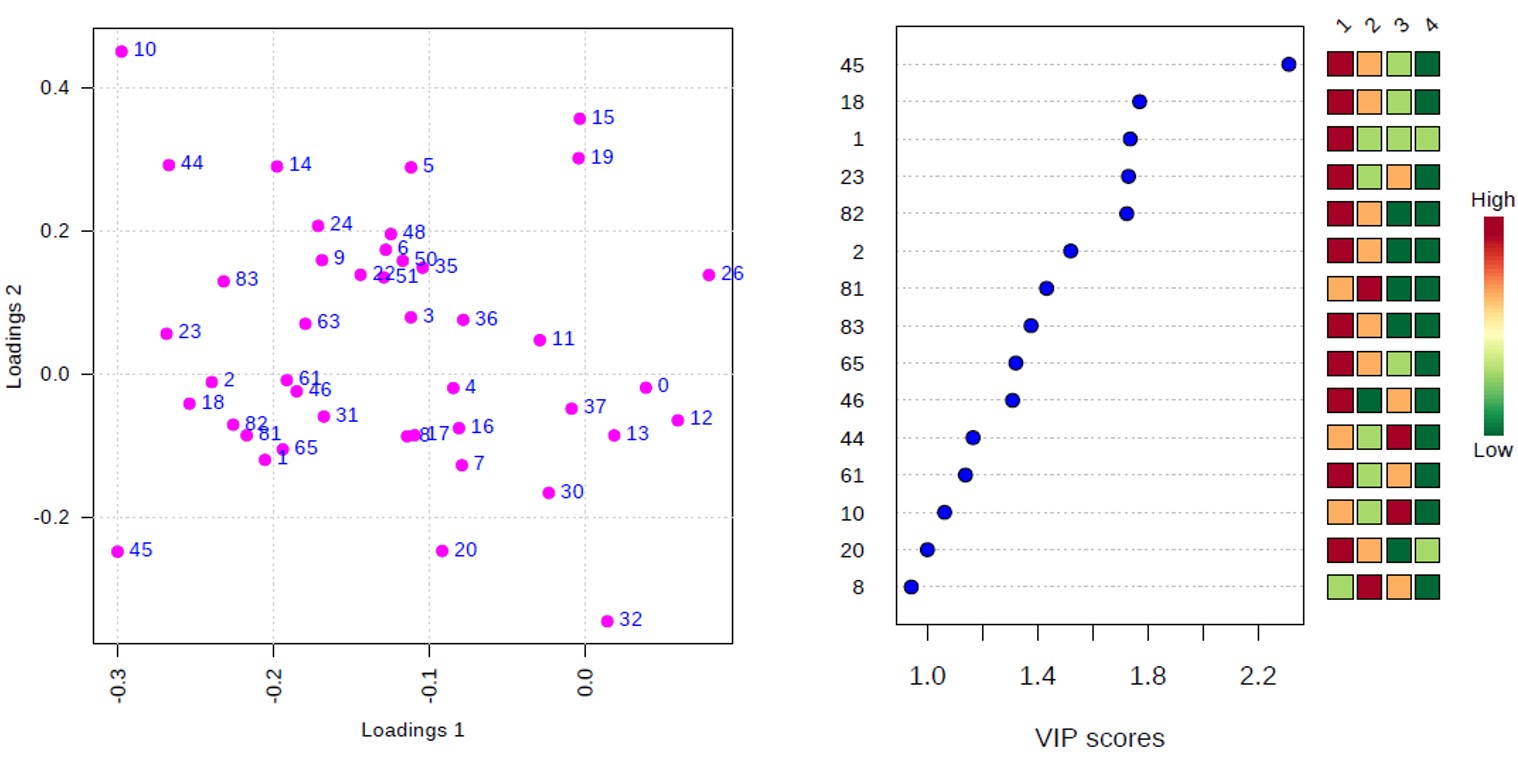

Supplement: Supplementary 3 — Supplementary Figure 3: PLS-DA loading plots (left) of the first two components for analysis of protein spots of salivary profiles from the different dog breeds. Variable importance in the projection (VIP) is presented (right). [file 7456894.f3.jpg]
